# Emerging Prognostic and Predictive Biomarkers for Human Cytomegalovirus Infection During Pregnancy: Unmet Needs and Future Perspectives

**DOI:** 10.3390/v17050705

**Published:** 2025-05-14

**Authors:** Salvatore Rotundo, Maria Teresa Tassone, Rosaria Lionello, Paolo Fusco, Francesca Serapide, Alessandro Russo

**Affiliations:** 1 Infectious Disease Unit, “San Giovanni di Dio” Hospital, 88900 Crotone, Italy; 2 Department of Medical and Surgical Sciences, “Magna Graecia” University, 88100 Catanzaro, Italy; mariateresatassone90@gmail.com (M.T.T.); f.serapide@unicz.it (F.S.); a.russo@unicz.it (A.R.); 3 Infectious and Tropical Disease Unit, “Renato Dulbecco” Teaching Hospital, 88100 Catanzaro, Italy; rosarialionello0@gmail.com (R.L.); paolofusco89@gmail.com (P.F.)

**Keywords:** biomarkers, HCMV, serology, DNAemia, NIPT, T-cell response, exosomes

## Abstract

Human cytomegalovirus (HCMV) infection during pregnancy is a leading cause of congenital infections worldwide, posing significant risks to fetal health. Despite advances in prenatal care, managing HCMV infection remains challenging. Early detection, accurate risk assessment, and timely intervention are critical to mitigating the adverse outcomes associated with congenital HCMV (cHCMV), such as neurodevelopmental delays and hearing loss. However, the current landscape of biomarkers for HCMV infection in pregnancy is marked by several unmet needs. These gaps in biomarker development and application limit our ability to predict fetal transmission, assess the risk of fetal damage, and prognosticate long-term outcomes. Addressing these challenges through the identification and validation of novel biomarkers could revolutionize the management of HCMV in pregnancy, leading to improved outcomes for both mothers and their children. This review examines the critical unmet needs regarding HCMV biomarkers during pregnancy, emphasizing the priority areas for further research and innovation.

## 1. Introduction

The pathogenesis of human cytomegalovirus (HCMV) infection during pregnancy is a complex interplay involving the virus, the maternal immune system, and the developing fetus. In pregnant women, HCMV infection typically begins when the virus enters through mucosal surfaces and starts replicating in epithelial cells. Later, the virus disseminates through the bloodstream. This primary infection usually presents as asymptomatic or with mild symptoms.

One of the most significant aspects of managing HCMV during pregnancy is the ability to predict and assess the severity of the infection in the fetus, which can be challenging given the virus’s ability to evade immune detection and persist in a latent form. Biomarkers could play a crucial role in overcoming these challenges. They offer the potential for non-invasive, earlier, and more accurate diagnosis, risk stratification, and monitoring of both maternal and fetal health.

Recent advancements in biomarker research have identified several promising candidates. Such biomarkers could enhance our ability to predict which fetuses are at higher risk for adverse outcomes and guide timely interventions. Understanding these emerging biomarkers is essential for improving the management of HCMV infection in pregnancy and mitigating its potential impact on neonatal health.

## 2. Pathogenesis

The pathogenesis of HCMV during pregnancy involves both the initial infection and subsequent reactivation of the virus, its impact on maternal and fetal immune responses, placental infection, and the potential for vertical transmission to the fetus [1]. The outcome of HCMV infection can vary widely, depending on the timing of infection, the maternal immune response, and other factors that affect both maternal and fetal health [2].

When a pregnant woman experiences a primary HCMV infection, the virus initially enters the body through mucosal surfaces, such as the oropharynx or genitals [3]. It begins replicating in epithelial cells and subsequently spreads throughout the body, establishing a systemic infection [4]. After this initial infection, HCMV can establish latency, primarily in myeloid progenitor cells, such as CD34+ cells and their CD14+ monocyte derivatives [4,5]. During latency, there is little viral gene expression, which is not able to lead to a productive infection, so the virus does not cause symptoms or signs of infection [6]. However, the virus can reactivate, especially during periods of immune suppression, such as pregnancy [3]. Reactivation leads to viral replication and can result in the transmission of HCMV to the fetus [3].

The maternal immune system responds to HCMV infection through both innate and adaptive mechanisms [7]. Innate immunity involves the activation of natural killer (NK) cells and the production of interferons, which play critical roles in suppressing viral replication [8]. In non-transmitting mothers, NK cells exhibited greater activity and maturity compared to transmitting mothers, suggesting a protective role of these cells in preventing HCMV transmission [9]. The adaptive immune response includes the generation of HCMV-specific T-cells and antibodies. The production of HCMV-specific antibodies, including IgM and IgG, occurs as part of this response [10]. IgG antibodies can cross the placenta, providing some degree of protection to the fetus, though this protection is not always sufficient to prevent fetal infection [11].

The placenta can be affected by HCMV in several ways [12]. The virus may infect the placenta directly through the bloodstream or by ascending from the cervix [12]. Within the placenta, HCMV replicates in trophoblast cells, which are crucial for implantation and the maintenance of pregnancy (the frequency of clinical sequalae in the fetus/newborn decreases with advancing gestational age and clinical sequalae are unlikely when maternal infection occurs in the second half of pregnancy). In a review of pooled data from 10 studies (796 fetuses), the rates of clinical sequelae (neurologic symptoms at birth or the termination of pregnancy because of CMV-associated findings in the central nervous system on ultrasonography or magnetic resonance imaging) by gestational age of maternal seroconversion were as follows:

• Periconceptional period (from four weeks before to six weeks after the last menstrual period)—28.8 percent (95% CI 2.4–55.1)

• First trimester—19.3 percent (95% CI 12.2–26.4)

• Second trimester—0.9 percent (95% CI 0–2.4)

• Third trimester—0.4 percent (95% CI 0–1.5) [13].

**Figure 1:**
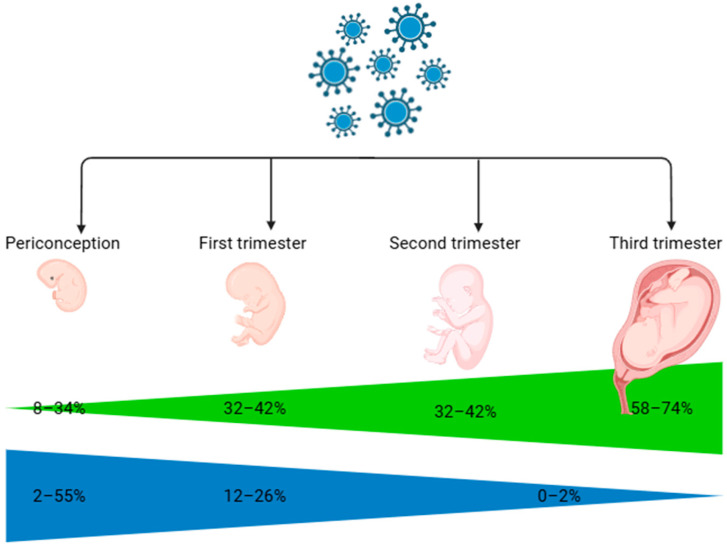
Rates of vertical transmission (green) and fetal disease (blue) for primary human cytomegalovirus infection in pregnancy.

The infection of these cells can disrupt their normal function, leading to impaired placental development and function [14]. Additionally, HCMV infection induces inflammation in the placenta, characterized by increased cytokine production and immune cell infiltration [15]. This inflammation can contribute to complications such as placental insufficiency and fetal growth restriction [16]. Fetal transmission of HCMV takes place when the virus crosses the placenta, leading to fetal infection. Primary HCMV infection during pregnancy carries a 30% to 40% risk of intrauterine transmission [17]. The risk of transmission is highest during a primary maternal infection [18] (Figure 1), particularly in the first trimester, though reactivation or reinfection with a different HCMV strain can also result in fetal infection.

Once infected, the fetus can experience a range of outcomes, from asymptomatic infection to severe congenital HCMV (cHCMV) disease. The virus can affect various fetal organs, including the brain, liver, and spleen, leading to potential complications such as microcephaly, intracranial calcifications, hearing loss, vision impairment, developmental delays, and other neurological deficits [3,17,19]. Among women with primary HCMV infection, 18% of their infants are symptomatic at birth, presenting with conditions such as jaundice, petechial rash, hepatosplenomegaly, and, in severe cases, death [20]. A classic study tracked these infants over time to assess long-term risks. Notably, up to 25% of those asymptomatic at birth developed sequelae within the first two years of life, including sensorineural hearing loss, cognitive deficits (with an IQ below 70), chorioretinitis, seizures, and death. Sequelae could manifest as late as 72 months later, with severe outcomes more likely in cases where the mother contracted the infection during the first half of pregnancy [21].

The fetal immune response to HCMV infection involves the production of cytokines and the initiation of immune responses [22]. However, the fetal immune system, which is still developing, may not effectively control the infection, resulting in extensive viral replication and tissue damage. In some cases, infants born with cHCMV may continue to have productive infection after birth, which can lead to long-term sequelae [23]. The virus can persist in various organs and tissues, potentially causing progressive hearing loss or developmental delays over time.

Genetic factors in both the mother and fetus can influence the severity of HCMV infection and the risk of adverse outcomes [24]. Additionally, co-infections or conditions that affect the maternal immune system may modify the course of HCMV infection during pregnancy, potentially impacting the severity of fetal infection and the risk of complications [25].

## 3. Limitations of Serological Diagnostics

Serological diagnostics, commonly used for screening HCMV infections, involve detecting IgM and IgG antibodies. However, this approach has various limitations, especially in the context of pregnancy, where understanding the timing of infection is crucial for assessing fetal risk. Complexities arise primarily from the nature of IgM and IgG antibodies themselves and the intricacies of interpreting their presence in a way that reliably differentiates between primary and reinfection from past exposure or reactivation. IgM antibodies are generally associated with recent infections but are prone to false positives in HCMV diagnostics, which may occur due to cross-reactivity with IgM from other herpesviruses or interference from rheumatoid factor or autoimmune disorders [26]. In many cases, IgM can remain elevated long after the initial infection or reappear during viral reactivations or reinfections [27,28]. This limitation complicates clinical decision making, often leading to unnecessary anxiety and additional testing.

In contrast, IgG antibodies typically indicate past exposure, but are less helpful in pinpointing recent infections [29]. While detecting HCMV antibodies confirms a prior infection, it does not indicate when the infection occurred, which is crucial during pregnancy. Although a 4-fold increase in IgG antibodies during pregnancy has been proposed as a prognostic biomarker for cHCMV infection [30], this approach requires knowledge of the patient’s serological status at the beginning of pregnancy, which is not always available. This is because it remains debated whether universal screening should be recommended for all women, as outlined by international guidelines [31]. In fact, most healthcare systems in various countries do not routinely recommend serological testing during pregnancy. The relevance of such screening should be assessed on a case-by-case basis in each country, considering local epidemiology and cost-effectiveness. For instance, a recent French cost-effectiveness study indicated that universal screening, combined with valaciclovir treatment, would be more cost-effective compared to current practices [32]. To clarify the timing of infection, clinicians usually use an IgG avidity test. Low avidity indicates a recent infection, while high avidity suggests an older, more established infection. However, this method must be interpreted with caution, as IgG avidity results are not always conclusive [33,34,35].

The reliance on both IgM and IgG markers for establishing the timing of a CMV infection further complicates the diagnostic picture. The overlapping nature of these biomarkers and their persistence can lead to uncertain interpretations, risking misclassification of the infection’s timing and type. This ambiguity is especially significant in pregnancy, where timing directly influences fetal transmission risk and potential outcomes [33,34].

Another crucial limitation of serological diagnostics in HCMV is their limited prognostic power regarding fetal risk. Although IgM and IgG serology indicate maternal infection, they do not provide specific insights into fetal involvement or the severity of potential cHCMV infection. Indeed, maternal IgM antibodies and IgG avidity tests, commonly used serological markers, have not been significantly associated with the risk of transmitting HCMV to the fetus [30]. For a more precise risk assessment, additional methods are often required to accurately evaluate fetal infection and prognosis. In summary, while serological diagnostics play a significant role in CMV detection, their limitations in accurately identifying recent infections, differentiating reactivations, and predicting fetal risk suggest the need for supplementary approaches.

## 4. Limitations of HCMV DNAemia

Quantitative polymerase chain reaction (qPCR) for HCMV DNA in the blood is a highly sensitive method for detecting HCMV DNAemia and is widely used to identify active viral replication. Its sensitivity is particularly high during the early stages of infection, when the viral load in the bloodstream tends to be elevated. However, qPCR does not provide information about the precise timing or type of infection—whether primary infection, reinfection, or reactivation. It detects only the presence of viral genomic material in the blood, which may reflect a productive infection or, in some cases, low-level reactivation from latent reservoirs such as myeloid progenitor cells. HCMV DNA may persist for variable periods, sometimes up to a year or longer, depending on individual immune responses, and this persistence complicates attempts to infer the timing of infection [31,36].

Therefore, qPCR testing in maternal blood is not a reliable standalone tool for determining the timing of HCMV infection or for assessing the specific risk of fetal transmission. An exception may occur in cases where HCMV-specific IgM antibodies are present without accompanying IgG seroconversion. In such scenarios, qPCR can help to clarify the clinical picture by confirming the presence of an active infection, suggesting recent acquisition, or alternatively ruling out ongoing replication—indicating that the IgM result may be a false positive, which is not uncommon. When interpreted in combination with serological findings, qPCR in whole blood can, thus, contribute to a more accurate assessment of maternal infection status [26].

The role of viral DNAemia during pregnancy as a prognostic biomarker for cHCMV transmission to the newborn is well-established [30,37]. The detection of HCMV DNA in maternal blood correlates with a significantly increased risk of vertical transmission, with studies indicating a 5- to 13-fold higher risk depending on the trimester of infection [30]. However, its value as a predictive biomarker remains uncertain. While qPCR and related DNA-based methods are routinely employed to monitor viral kinetics during antiviral therapy [38], few studies have specifically investigated the predictive role of maternal HCMV DNAemia in treatment response or neonatal outcomes during pregnancy. Further research is warranted to clarify how maternal DNAemia could guide therapeutic decisions or predict vertical transmission risk.

## 5. The Role of HCMV DNA in Amniotic Fluid Samples and Urine Samples

qPCR for HCMV DNA in amniotic fluid samples is considered in current clinical practice as a mandatory diagnostic test to exclude fetal infection and is performed after approximately 20 weeks plus one day of gestation, taking care that the suspected primary infection occurred no earlier than 8 weeks [39]. Traditionally, sensitivity has been reported to be highest after 21 weeks of gestation; however, emerging data suggest that an interval of 8 weeks following maternal primary infection is a more appropriate benchmark. In a prospective study of maternal valacyclovir treatment for the secondary prevention of fetal infection, amniocentesis for fetal infection diagnosis performed between 17 and 20 weeks, at least 8 weeks from maternal primary infection, had a sensitivity of 95.8% (95% CI 79.8–99.8 percent), specificity of 100% (95% CI 91.8–100.0), positive predictive value of 100% (95% CI 85.7–100.0), and negative predictive value of 97.7% (95% CI 88.2–99.9) [40]. In a retrospective study that reviewed 264 pregnancies at 17 to 23 weeks of gestation with at least 8 weeks between seroconversion and amniocentesis, the diagnostic sensitivity was similar before versus after 21 weeks (87.2% and 92.1%, respectively), as was the negative predictive value (93.6% and 96.8%, respectively) [41]. The first 1 mL of fluid obtained should be discarded to reduce the risk of maternal cell contamination [41,42,43]. Rarely, false-positive results occur from contamination of the amniotic fluid sample by maternal fluids.

Regarding the viral load in amniotic fluid, the prognostic value of the CMV viral load in this sample has been studied as a possible predictor of symptomatic disease at birth for fetuses with normal ultrasound [44,45,46]. One study found that a viral load of >100,000 copies/mL of amniotic fluid at weeks 21 to 23 of gestation distinguished 7 fetuses/newborns with signs and/or symptoms of congenital CMV infection from 16 who appeared normal and asymptomatic [44]. Another retrospective study calculated a negative predictive value for symptoms at birth or at termination of pregnancy of 93% for ultrasound alone versus 95% for ultrasound and viral load in amniotic fluid [45]. However, the viral load threshold for predicting symptoms at birth has not been robustly defined or validated in independent prospective cohorts. Also, CMV DNA in maternal urine samples could be a valuable marker, particularly to predict fetal infection. In fact, in a study performed on 150 pregnant women with proven CMV primary infection, a statistically significant association was found between the presence of CMV in maternal urine and newborn infection (OR 2.03 95%CI 1.03–3.99), and taking into consideration those samples that were positive for CMV in maternal urine, the median viral load value was significantly higher in those patients who transmitted to their offspring [47].

## 6. HCMV T-Cell Immunity as a Prognostic Biomarker

During pregnancy, the immune response undergoes significant and delicate modifications that establish a critical balance. On the one hand, the maternal immune system must adapt to protect the developing fetus, which is genetically distinct from the mother. This adaptation involves a shift towards a more immunotolerant state, allowing the fetus to develop without being attacked by maternal immune cells [48]. On the other hand, it remains essential for the mother’s immune system to maintain its ability to effectively defend against pathogens [49]. Consequently, the success of pregnancy relies on this finely tuned interplay between tolerance and immune defense, which is crucial for both maternal and fetal health [50].

In recent decades, the adaptive immune response, particularly the T-cell response, has gained increasing attention as a biomarker both for prognostic and predictive purposes in the field of infectious diseases [37,51,52,53,54]. The peripheral HCMV T-cell response, being non-invasive and less risky than amniocentesis, offers a promising predictive diagnostic tool for cHCMV infection [55]. Although current T-cell-response-based assays still face limitations in specificity—since the detection of a T-cell response does not necessarily distinguish between recent and past infection—they remain a promising tool, because they provide functional insight into the host immune response that cannot be obtained through serology or PCR alone. Importantly, while both primary infection and reactivation can lead to congenital HCMV transmission, the ability to differentiate between them is clinically relevant, as primary infection is associated with a significantly higher risk of symptomatic congenital disease. T-cell immunity assays may help to support this distinction when interpreted alongside serological and molecular findings, although further standardization and validation are needed [31].

In a study evaluating the use of HCMV-specific enzyme-linked immunospot (ELISpot) and interferon gamma-releasing assay (IGRA) as biomarkers for cHCMV risk in 80 pregnant women with primary or non-primary HCMV infections, it was found that high HCMV ELISpot levels correlated with increased fetal transmission risk, while IGRA tests did not. Additionally, cHCMV risk was associated with low HCMV IgG avidity and positive maternal viremia and viruria [53].

In a prospective study conducted across eight Spanish hospitals, 135 pregnant women with primary HCMV infections were evaluated to assess if maternal HCMV-specific T-cell responses correlated with fetal transmission. In this study, no association between HCMV-specific T-cell responses (CD4+ and CD8+) and fetal transmission risk was found. However, they identified that women with undetectable HCMV viral loads at diagnosis did not transmit the virus, suggesting that detectable viremia might serve as a transmission predictor. Therefore, this study emphasized that T-cell response alone may not be a reliable marker for HCMV transmission and highlighted the need for combined indicators, including viral load [37]. Accordingly, one study investigated the role of CMV-specific immune responses in predicting congenital CMV transmission, focusing on CMV IgG avidity and CMV ELISpot assays. It found that the combined use of CMV IgG avidity and high CMV ELISpot demonstrated a superior predictive accuracy compared to either test alone. The study also highlighted the potential link between strong cell-mediated immune responses and congenital transmission, suggesting that prolonged maternal viremia or immune-mediated placental changes may facilitate viral transmission, emphasizing the relevance of CMV-specific immunity in diagnosing cHCMV infection and informing future management strategies [55].

Taken together, these studies suggest that ELISspot may have a superior prognostic power as a biomarker for fetal infection risk, possibly because it assesses both CD4+ and CD8+ T-cell responses, unlike the IGRA test, which targets only CD8+ responses [53]. Moreover, an increased cHCMV rate when low HCMV IgG avidity and a strong maternal T-cell response to HCMV were present suggested that congenital CMV may result from an imbalanced Th1/Th2 immune response to HCMV [53,55]. In this context, while a robust T-cell response is generally seen as beneficial for controlling infections, it may paradoxically lead to an increased risk of cHCMV due to sustained viral activity and inflammation. The resulting inflammatory environment could disrupt normal placental function and facilitate viral transmission, emphasizing the complexity of immune responses during pregnancy and their potential implications for fetal health [37]. In line with this observation, another study introduced a novel “relative response” (RR) method to measure individual IFN-γ responses to HCMV, aiming to predict fetal HCMV transmission in pregnant women with primary infections. This normalization technique could serve as a prognostic tool, potentially outperforming conventional immune assessments by accounting for individual immune variability. A lower IFN-γ RR (<1.8%) was significantly associated with a reduced transmission risk, lowering the probability from 40% to 8% [54].

Interestingly, a recent observational study evaluated the utility of monitoring plasma ganciclovir levels in predicting HCMV DNA clearance in 13 allogeneic stem cell transplant recipients receiving preemptive treatment with intravenous ganciclovir or oral valganciclovir. The findings indicated that monitoring trough plasma ganciclovir levels did not reliably correlate with therapeutic response. In contrast, immunological monitoring, specifically the assessment of pp65 and immediate-early (IE)-1-specific IFN-γ-producing CD8+ T-cells, demonstrated a more significant relationship with HCMV clearance [56]. Applying this concept to pregnancy, immunological monitoring could serve as a valuable tool for assessing maternal immune responses to HCMV during antiviral treatment, potentially guiding therapeutic decisions. However, translating these findings into clinical practice would require additional research to validate specific T-cell responses as predictive biomarkers for HCMV treatment outcomes during pregnancy.

In summary, although several studies have explored the relationship between maternal immune responses and fetal transmission risk, findings have been inconsistent. Some studies suggest that high levels of HCMV-specific T-cell responses correlate with an increased risk of fetal transmission, while others emphasize the importance of viral load in predicting transmission. While T-cell responses, especially those measured by ELISpot, show promise in predicting fetal HCMV transmission, a comprehensive approach that includes immune response profiling and viral load measurements will be essential for accurate risk assessment. Further studies are needed to clarify the role of specific immune responses in predicting cHCMV infection and to develop more reliable biomarkers to guide clinical decision making.

## 7. Non-Invasive Prenatal Testing

The use of Non-Invasive Prenatal Testing (NIPT) represents a significant innovation in clinical practice, providing a safe and accurate method for analyzing fetal DNA present in maternal blood. This technique allows for the identification of genetic anomalies and hereditary conditions without the risks associated with invasive procedures such as amniocentesis [57]. Additionally, NIPT has emerged as the most promising technique for managing HCMV infections during pregnancy, enabling the early detection and monitoring of this virus [58,59]. Two large cohort studies examined NIPT for the early detection of HCMV DNA in pregnancy to improve prenatal care and reduce associated risks. In a study by Tong et al. [59], both the study and control groups consisted of pregnant women undergoing NIPT. However, the authors did not distinguish groups based on HCMV serostatus (i.e., seronegative vs. seropositive). Instead, they retrospectively analyzed microbial cfDNA—including HCMV DNA—across a very large cohort of 107,763 samples to define a reference baseline. Notably, HCMV DNA was detected in approximately 0.05% of the total population, indicating its presence even among healthy, asymptomatic individuals. Nevertheless, the study did not investigate genetic polymorphisms or host genomic associations with viral detection, and no data were provided regarding polymorphism analysis. Therefore, while the findings demonstrated the low-level detection of HCMV DNA across the population, they do not allow for inference about infection status, serology, or host susceptibility. Another multicenter study [58], which included the largest cohort of pregnant women published to date, demonstrated that in pregnancies testing positive for cell-free DNA (cfDNA)-HCMV, the fragment prevalence metric correlated with active HCMV infections, either recent primary infections or reactivated past infections. In contrast, cfDNA-HCMV-negative samples corresponded with negative qPCR results and serological profiles indicating no recent HCMV infections, suggesting the utility of cfDNA-HCMV analysis as a screening method to identify pregnancies at risk for maternal–fetal viral transmission. The study found an overall frequency of 0.94% of cfDNA samples containing HCMV fragments among 204,818 participants, which is significantly higher than the 0.40% previously reported in Chinese populations [59]. This discrepancy may arise from differences in detection methods or true population variances. Validation through HCMV-qPCR confirmed that all cfDNA-HCMV-negative samples were qPCR-negative. However, 67.8% of cfDNA-HCMV-positive samples were also qPCR-negative, indicating the qPCR method’s limitations in detecting very low viral loads. In cfDNA-HCMV-negative samples, serological testing indicated that 59.1% were seronegative, while 40.9% showed evidence of past infections. In contrast, serology from cfDNA-HCMV-positive samples suggested recent primary infections in 28.6% of cases, while two cfDNA-HCMV-positive samples were seronegative, implying very recent infections not yet detectable by serology [58].

In summary, these findings support NIPT as a promising, non-invasive method for early HCMV detection, offering not only genetic assessment, but also an opportunity for understanding the interactions between viruses and host genetic variability, paving the way for new avenues in biomedical research and preventive medicine.

## 8. The Possible Role of Exosomes in HCMV Infections

Exosomes, small membrane-bound vesicles released by cells, have a complex and emerging role in several viral infections [60,61,62]. These vesicles serve multiple functions, including transporting immune-stimulating molecules, presenting antigens, and activating immune cells. They also carry infection-specific biomarkers, making them valuable for diagnostic purposes, while offering potential as drug delivery vehicles [63]. In pregnancy, exosomes released by the placenta are involved in essential physiological exchanges, including immune regulation, that support fetal development and protect against infections [63]. Therefore, exosomes are being explored as diagnostic biomarkers for infectious diseases. The role of exosomes in HCMV infection during pregnancy is a growing area of interest, as these small extracellular vesicles hold significant promise both as biomarkers and as key players in the communication between the placenta, mother, and fetus [64]. In a study analyzing exosomes derived from first-trimester placentas, the function and composition of exosomes were found to be notably altered in the context of HCMV infection [65]. These changes can have both positive and negative implications for pregnancy [65,66]. Placenta-derived exosomes may serve as non-invasive biomarkers, providing information about the health of both the fetus and the placenta during pregnancy [67]. They play a fundamental role in the communication between the placenta and the mother, as well as the fetus, facilitating the exchange of essential signals for immune regulation and placental development [68]. Specifically, in HCMV infection, exosomes maintain their integrity and show variations in surface markers, which can be used to identify and monitor ongoing infection [65]. During HCMV infection, the composition of exosomes undergoes significant alterations, which may impact their protective and regulatory roles. These changes could have detrimental effects on placental function and fetal development. Specifically, modifications in the surface markers of exosomes may enhance the ability of the virus to spread within placental tissue, exacerbating the infection. This disruption not only worsens the viral load within the placenta, but also elevates the risk of fetal damage, potentially leading to complications such as congenital infection, developmental delays, or other long-term health issues for the fetus.

However, despite their potential, there is a significant gap in research exploring the role of exosomes during pregnancy, particularly in relation to HCMV infections. Future studies should focus on gaining a deeper understanding of the specific role that exosomes play in pregnancy, especially in the context of HCMV infection. This would help to unlock their potential as non-invasive diagnostic tools and therapeutic targets.

**Figure 2:**
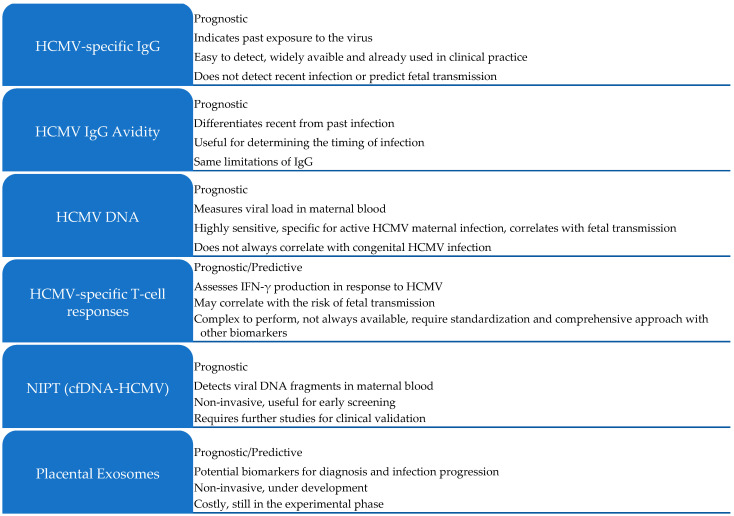
Biomarkers for human cytomegalovirus (HCMV) infection in pregnancy. Overview of potential biomarkers used for diagnosing, monitoring, and predicting the outcomes of HCMV infection during pregnancy. These include traditional serological and molecular markers (HCMV DNA, IgG, and IgG avidity), immune response markers (T-cell responses), and emerging non-invasive biomarkers such as non-invasive prenatal testing (NIPT) for cell-free DNA (cfDNA-HCMV) and placental exosomes.

A summary of the emerging biomarkers discussed in this review is presented in Figure 2.

## 9. Unmet Needs and Future Perspectives

Despite advancements in the understanding of HCMV infection during pregnancy, several critical gaps remain in biomarker development, therapeutic monitoring, and risk stratification. The current lack of validated predictive biomarkers hinders the ability to effectively tailor antiviral treatment strategies. The introduction of valaciclovir therapy for pregnant women represents a milestone in HCMV management [69]. However, a key unmet need is the development of predictive biomarkers that can assess treatment efficacy, detect early signs of drug resistance, and anticipate adverse effects. Given the emergence of antiviral resistance in prolonged antiviral treatment [70], the identification of resistance-associated mutations in circulating HCMV DNA could guide clinical decisions and prevent treatment failure. While maternal DNAemia has been established as a prognostic marker for vertical transmission, its predictive value for treatment response and fetal outcomes remains unclear. Future studies should focus on identifying biomarkers capable of distinguishing pregnancies that would benefit most from antiviral therapy, optimizing timing, and reducing unnecessary drug exposure.

NIPT for cfDNA-HCMV has shown promise in detecting fetal infection earlier than traditional methods. However, the standardization of NIPT for HCMV screening, along with defining its clinical thresholds for intervention, remains an essential area for future research. Similarly, the role of exosome-derived biomarkers in monitoring immune responses and placental involvement in HCMV infection is an emerging field that warrants further exploration.

In conclusion, the integration of novel predictive biomarkers, personalized treatment approaches, and innovative non-invasive diagnostics will be crucial in improving the management of HCMV infection during pregnancy. Future research should focus on refining these strategies to optimize maternal and fetal outcomes while minimizing unnecessary interventions. In recent years, valaciclovir therapy has emerged as a standard in the management of HCMV infections during pregnancy.

## Data Availability

Not applicable.

## Abbreviations

The following abbreviations are used in this manuscript:

HCMV	Human Cytomegalovirus
cHCMV	Congenital Human Cytomegalovirus
NK	Natural Killer
IgM	Immunoglobulin M
IgG	Immunoglobulin G
PCR	Polymerase Chain Reaction
IE-1	Immediate-Early 1
ELISpot	Enzyme-Linked Immunospot Assay
IGRA	Interferon-Gamma Release Assay
IFN-γ	Interferon-Gamma
RR	Relative Response
NIPT	Non-Invasive Prenatal Testing
cfDNA	Cell-Free DNA
qPCR	Quantitative Polymerase Chain Reaction

## References

[1] Pass R.F., Anderson B. (2014). Mother-to-Child Transmission of Cytomegalovirus and Prevention of Congenital Infection. J. Pediatr. Infect. Dis. Soc..

[2] Fowler K.B., Stagno S., Pass R.F. (2003). Maternal immunity and prevention of congenital cytomegalovirus infection. JAMA.

[3] Pontes K.F.M., Nardozza L.M.M., Peixoto A.B., Werner H., Tonni G., Granese R., Araujo Junior E. (2024). Cytomegalovirus and Pregnancy: A Narrative Review. J. Clin. Med..

[4] Mihalic A., Zeleznjak J., Lisnic B., Jonjic S., Juranic Lisnic V., Brizic I. (2024). Immune surveillance of cytomegalovirus in tissues. Cell. Mol. Immunol..

[5] De Groof T.W.M., Elder E.G., Lim E.Y., Heukers R., Bergkamp N.D., Groves I.J., Wills M., Sinclair J.H., Smit M.J. (2021). Targeting the latent human cytomegalovirus reservoir for T-cell-mediated killing with virus-specific nanobodies. Nat. Commun..

[6] Muller L., Di Benedetto S. (2024). Immunosenescence and Cytomegalovirus: Exploring Their Connection in the Context of Aging, Health, and Disease. Int. J. Mol. Sci..

[7] La Rosa C., Diamond D.J. (2012). The immune response to human CMV. Future Virol..

[8] Zuo W., Zhao X. (2021). Natural killer cells play an important role in virus infection control: Antiviral mechanism, subset expansion and clinical application. Clin. Immunol..

[9] Pighi C., Rotili A., De Luca M., Chiurchiu S., Calo Carducci F.I., Rossetti C., Cifaldi L., Bei R., Caforio L., Bernardi S. (2024). Characterization of Natural Killer Cell Profile in a Cohort of Infected Pregnant Women and Their Babies and Its Relation to CMV Transmission. Viruses.

[10] Picarda G., Benedict C.A. (2018). Cytomegalovirus: Shape-Shifting the Immune System. J. Immunol..

[11] Langel S.N., Blasi M., Permar S.R. (2022). Maternal immune protection against infectious diseases. Cell Host Microbe.

[12] Megli C.J., Coyne C.B. (2022). Infections at the maternal-fetal interface: An overview of pathogenesis and defence. Nat. Rev. Microbiol..

[13] Cruz-Holguin V.J., Gonzalez-Garcia L.D., Velazquez-Cervantes M.A., Arevalo-Romero H., De Jesus-Gonzalez L.A., Helguera-Repetto A.C., Leon-Reyes G., Salazar M.I., Cedillo-Barron L., Leon-Juarez M. (2024). Collateral Damage in the Placenta during Viral Infection in Pregnancy: A Possible Mechanism for Vertical Transmission and an Adverse Pregnancy Outcome. Diseases.

[14] Weckman A.M., Ngai M., Wright J., McDonald C.R., Kain K.C. (2019). The Impact of Infection in Pregnancy on Placental Vascular Development and Adverse Birth Outcomes. Front. Microbiol..

[15] Chudnovets A., Liu J., Narasimhan H., Liu Y., Burd I. (2020). Role of Inflammation in Virus Pathogenesis during Pregnancy. J. Virol..

[16] Tsikouras P., Antsaklis P., Nikolettos K., Kotanidou S., Kritsotaki N., Bothou A., Andreou S., Nalmpanti T., Chalkia K., Spanakis V. (2024). Diagnosis, Prevention, and Management of Fetal Growth Restriction (FGR). J. Pers. Med..

[17] Stagno S., Pass R.F., Cloud G., Britt W.J., Henderson R.E., Walton P.D., Veren D.A., Page F., Alford C.A. (1986). Primary cytomegalovirus infection in pregnancy. Incidence, transmission to fetus, and clinical outcome. JAMA.

[18] Chatzakis C., Ville Y., Makrydimas G., Dinas K., Zavlanos A., Sotiriadis A. (2020). Timing of primary maternal cytomegalovirus infection and rates of vertical transmission and fetal consequences. Am. J. Obstet. Gynecol..

[19] Davis N.L., King C.C., Kourtis A.P. (2017). Cytomegalovirus infection in pregnancy. Birth Defects Res..

[20] Fowler K.B., Stagno S., Pass R.F., Britt W.J., Boll T.J., Alford C.A. (1992). The outcome of congenital cytomegalovirus infection in relation to maternal antibody status. N. Engl. J. Med..

[21] Pass R.F., Fowler K.B., Boppana S.B., Britt W.J., Stagno S. (2006). Congenital cytomegalovirus infection following first trimester maternal infection: Symptoms at birth and outcome. J. Clin. Virol..

[22] Brizic I., Hirsl L., Britt W.J., Krmpotic A., Jonjic S. (2018). Immune responses to congenital cytomegalovirus infection. Microbes Infect..

[23] Auriti C., De Rose D.U., Santisi A., Martini L., Piersigilli F., Bersani I., Ronchetti M.P., Caforio L. (2021). Pregnancy and viral infections: Mechanisms of fetal damage, diagnosis and prevention of neonatal adverse outcomes from cytomegalovirus to SARS-CoV-2 and Zika virus. Biochim. Biophys. Acta Mol. Basis Dis..

[24] Pass R.F., Arav-Boger R. (2018). Maternal and fetal cytomegalovirus infection: Diagnosis, management, and prevention. F1000Research.

[25] Britt W.J. (2018). Maternal Immunity and the Natural History of Congenital Human Cytomegalovirus Infection. Viruses.

[26] Saldan A., Forner G., Mengoli C., Gussetti N., Palu G., Abate D. (2017). Testing for Cytomegalovirus in Pregnancy. J. Clin. Microbiol..

[27] Kitamura A., Toriyabe K., Hagimoto-Akasaka M., Hamasaki-Shimada K., Ikejiri M., Minematsu T., Suga S., Kondo E., Kihira M., Morikawa F. (2023). Revision of Cytomegalovirus Immunoglobulin M Antibody Titer Cutoff in a Maternal Antibody Screening Program in Japan: A Cohort Comparison Involving a Total of 32,000 Pregnant Women. Viruses.

[28] Revello M.G., Gerna G. (2002). Diagnosis and management of human cytomegalovirus infection in the mother, fetus, and newborn infant. Clin. Microbiol. Rev..

[29] Carlson A., Norwitz E.R., Stiller R.J. (2010). Cytomegalovirus infection in pregnancy: Should all women be screened?. Rev. Obstet. Gynecol..

[30] Huang Y., Tang J., Yu H., Song Q., Hao M., Wang H., Liu J., Dong Y., Liang M., Zhuang S. (2024). Reconsideration of Maternal Serological Testing for Predicting Congenital CMV Infection. J. Infect. Dis..

[31] Leruez-Ville M., Chatzakis C., Lilleri D., Blazquez-Gamero D., Alarcon A., Bourgon N., Foulon I., Fourgeaud J., Gonce A., Jones C.E. (2024). Consensus recommendation for prenatal, neonatal and postnatal management of congenital cytomegalovirus infection from the European congenital infection initiative (ECCI). Lancet Reg. Health Eur..

[32] Perillaud-Dubois C., Hachicha-Maalej N., Lepers C., Letamendia E., Teissier N., Cousien A., Sibiude J., Deuffic-Burban S., Vauloup-Fellous C., Picone O. (2023). Cost-effectiveness of screening and valacyclovir-based treatment strategies for first-trimester cytomegalovirus primary infection in pregnant women in France. Ultrasound Obstet. Gynecol..

[33] Eggers M., Bader U., Enders G. (2000). Combination of microneutralization and avidity assays: Improved diagnosis of recent primary human cytomegalovirus infection in single serum sample of second trimester pregnancy. J. Med. Virol..

[34] Rawlinson W.D., Boppana S.B., Fowler K.B., Kimberlin D.W., Lazzarotto T., Alain S., Daly K., Doutre S., Gibson L., Giles M.L. (2017). Congenital cytomegalovirus infection in pregnancy and the neonate: Consensus recommendations for prevention, diagnosis, and therapy. Lancet Infect. Dis..

[35] Revello M.G., Genini E., Gorini G., Klersy C., Piralla A., Gerna G. (2010). Comparative evaluation of eight commercial human cytomegalovirus IgG avidity assays. J. Clin. Virol..

[36] Gault E., Michel Y., Dehee A., Belabani C., Nicolas J.C., Garbarg-Chenon A. (2001). Quantification of human cytomegalovirus DNA by real-time PCR. J. Clin. Microbiol..

[37] Soriano-Ramos M., Esquivel-De la Fuente E., Albert Vicent E., de la Calle M., Baquero-Artigao F., Dominguez-Rodriguez S., Cabanes M., Gomez-Montes E., Gonce A., Valdes-Bango M. (2023). The role of the T-cell mediated immune response to Cytomegalovirus infection in intrauterine transmission. PLoS ONE.

[38] Weinberger S., Steininger C. (2022). Reliable quantification of Cytomegalovirus DNAemia in Letermovir treated patients. Antivir. Res..

[39] Gazzetta Ufficiale. https://www.gazzettaufficiale.it/atto/serie_generale/caricaDettaglioAtto/originario?atto.dataPubblicazioneGazzetta=2020-12-30&atto.codiceRedazionale=20A07138&elenco30giorni=true.

[40] Faure-Bardon V., Fourgeaud J., Stirnemann J., Leruez-Ville M., Ville Y. (2021). Secondary prevention of congenital cytomegalovirus infection with valacyclovir following maternal primary infection in early pregnancy. Ultrasound Obstet. Gynecol..

[41] Enders M., Daiminger A., Exler S., Enders G. (2017). Amniocentesis for prenatal diagnosis of cytomegalovirus infection: Challenging the 21 weeks’ threshold. Prenat. Diagn..

[42] Guerra B., Lazzarotto T., Quarta S., Lanari M., Bovicelli L., Nicolosi A., Landini M.P. (2000). Prenatal diagnosis of symptomatic congenital cytomegalovirus infection. Am. J. Obstet. Gynecol..

[43] Liesnard C., Donner C., Brancart F., Gosselin F., Delforge M.L., Rodesch F. (2000). Prenatal diagnosis of congenital cytomegalovirus infection: Prospective study of 237 pregnancies at risk. Obstet. Gynecol..

[44] Lazzarotto T., Varani S., Guerra B., Nicolosi A., Lanari M., Landini M.P. (2000). Prenatal indicators of congenital cytomegalovirus infection. J. Pediatr..

[45] Leruez-Ville M., Stirnemann J., Sellier Y., Guilleminot T., Dejean A., Magny J.F., Couderc S., Jacquemard F., Ville Y. (2016). Feasibility of predicting the outcome of fetal infection with cytomegalovirus at the time of prenatal diagnosis. Am. J. Obstet. Gynecol..

[46] Ornoy A., Diav-Citrin O. (2006). Fetal effects of primary and secondary cytomegalovirus infection in pregnancy. Reprod. Toxicol..

[47] Delforge M.L., Costa E., Brancart F., Goldman D., Montesinos I., Zaytouni S., Marchant A., Donner C. (2017). Presence of Cytomegalovirus in urine and blood of pregnant women with primary infection might be associated with fetal infection. J. Clin. Virol..

[48] Abu-Raya B., Michalski C., Sadarangani M., Lavoie P.M. (2020). Maternal Immunological Adaptation During Normal Pregnancy. Front. Immunol..

[49] Rackaityte E., Halkias J. (2020). Mechanisms of Fetal T Cell Tolerance and Immune Regulation. Front. Immunol..

[50] Yang F., Zheng Q., Jin L. (2019). Dynamic Function and Composition Changes of Immune Cells During Normal and Pathological Pregnancy at the Maternal-Fetal Interface. Front. Immunol..

[51] Rotundo S., Vecchio E., Abatino A., Giordano C., Mancuso S., Tassone M.T., Costa C., Russo A., Trecarichi E.M., Cuda G. (2022). Spike-specific T-cell responses in patients with COVID-19 successfully treated with neutralizing monoclonal antibodies against SARS-CoV-2. Int. J. Infect. Dis..

[52] Rotundo S., Tassone M.T., Serapide F., Russo A., Trecarichi E.M. (2024). Incipient tuberculosis: A comprehensive overview. Infection.

[53] Forner G., Saldan A., Mengoli C., Gussetti N., Palu G., Abate D. (2016). Cytomegalovirus (CMV) Enzyme-Linked Immunosorbent Spot Assay but Not CMV QuantiFERON Assay Is a Novel Biomarker To Determine Risk of Congenital CMV Infection in Pregnant Women. J. Clin. Microbiol..

[54] Eldar-Yedidia Y., Bar-Meir M., Hillel M., Abitbol G., Broide E., Falk R., Assous M., Schlesinger Y. (2016). Low Interferon Relative-Response to Cytomegalovirus Is Associated with Low Likelihood of Intrauterine Transmission of the Virus. PLoS ONE.

[55] Saldan A., Forner G., Mengoli C., Gussetti N., Palu G., Abate D. (2015). Strong Cell-Mediated Immune Response to Human Cytomegalovirus Is Associated With Increased Risk of Fetal Infection in Primarily Infected Pregnant Women. Clin. Infect. Dis..

[56] Gimenez E., Solano C., Azanza J.R., Amat P., Navarro D. (2014). Monitoring of trough plasma ganciclovir levels and peripheral blood cytomegalovirus (CMV)-specific CD8+ T cells to predict CMV DNAemia clearance in preemptively treated allogeneic stem cell transplant recipients. Antimicrob. Agents Chemother..

[57] Abedalthagafi M., Bawazeer S., Fawaz R.I., Heritage A.M., Alajaji N.M., Faqeih E. (2023). Non-invasive prenatal testing: A revolutionary journey in prenatal testing. Front. Med..

[58] Faas B.H.W., Astuti G., Melchers W.J.G., Reuss A., Gilissen C., Macville M.V.E., Ghesquiere S.A.I., Houben L.M.H., Srebniak M.I., Geeven G. (2024). Early detection of active Human CytomegaloVirus (hCMV) infection in pregnant women using data generated for noninvasive fetal aneuploidy testing. eBioMedicine.

[59] Tong X., Yu X., Du Y., Su F., Liu Y., Li H., Liu Y., Mu K., Liu Q., Li H. (2022). Peripheral Blood Microbiome Analysis via Noninvasive Prenatal Testing Reveals the Complexity of Circulating Microbial Cell-Free DNA. Microbiol. Spectr..

[60] Mimmi S., Zimbo A.M., Rotundo S., Cione E., Nistico N., Aloisio A., Maisano D., Tolomeo A.M., Dattilo V., Lionello R. (2023). SARS CoV-2 spike protein-guided exosome isolation facilitates detection of potential miRNA biomarkers in COVID-19 infections. Clin. Chem. Lab. Med..

[61] Chen J., Li C., Li R., Chen H., Chen D., Li W. (2021). Exosomes in HIV infection. Curr. Opin. HIV AIDS.

[62] Gheitasi H., Sabbaghian M., Shekarchi A.A., Mirmazhary A.A., Poortahmasebi V. (2024). Exosome-mediated regulation of inflammatory pathway during respiratory viral disease. Virol. J..

[63] Kalluri R., LeBleu V.S. (2020). The biology, function, and biomedical applications of exosomes. Science.

[64] Ghafourian M., Mahdavi R., Akbari Jonoush Z., Sadeghi M., Ghadiri N., Farzaneh M., Mousavi Salehi A. (2022). The implications of exosomes in pregnancy: Emerging as new diagnostic markers and therapeutics targets. Cell Commun. Signal..

[65] Bergamelli M., Martin H., Benard M., Ausseil J., Mansuy J.M., Hurbain I., Mouysset M., Groussolles M., Cartron G., Tanguy le Gac Y. (2021). Human Cytomegalovirus Infection Changes the Pattern of Surface Markers of Small Extracellular Vesicles Isolated From First Trimester Placental Long-Term Histocultures. Front. Cell Dev. Biol..

[66] Kaminski V.L., Ellwanger J.H., Chies J.A.B. (2019). Extracellular vesicles in host-pathogen interactions and immune regulation—Exosomes as emerging actors in the immunological theater of pregnancy. Heliyon.

[67] Czernek L., Duchler M. (2020). Exosomes as Messengers Between Mother and Fetus in Pregnancy. Int. J. Mol. Sci..

[68] Jin J., Menon R. (2018). Placental exosomes: A proxy to understand pregnancy complications. Am. J. Reprod. Immunol..

[69] Shahar-Nissan K., Pardo J., Peled O., Krause I., Bilavsky E., Wiznitzer A., Hadar E., Amir J. (2020). Valaciclovir to prevent vertical transmission of cytomegalovirus after maternal primary infection during pregnancy: A randomised, double-blind, placebo-controlled trial. Lancet.

[70] Acquier M., Taton B., Alain S., Garrigue I., Mary J., Pfirmann P., Visentin J., Hantz S., Merville P., Kaminski H. (2023). Cytomegalovirus DNAemia Requiring (Val)Ganciclovir Treatment for More Than 8 Weeks Is a Key Factor in the Development of Antiviral Drug Resistance. Open Forum Infect. Dis..

